# Novel coronavirus 2019 (COVID-19): A case report and review of treatments

**DOI:** 10.1097/MD.0000000000020207

**Published:** 2020-05-08

**Authors:** Steven Douedi, Jeffrey Miskoff

**Affiliations:** a Department of Medicine, Hackensack Meridian Jersey Shore University Medical Center Neptune; b Department of Medicine, Hackensack Meridian School of Medicine at Seton Hall University Nutley; c Department of Pulmonology and Critical Care, Hackensack Meridian Jersey Shore University Medical Center Neptune, NJ, USA.

**Keywords:** acute respiratory distress syndrome, coronavirus, novel coronavirus 2019, infection, respiratory, severe acute respiratory syndrome coronavirus 2

## Abstract

**Rationale:**

Novel coronavirus 2019 (COVID-19) also known as severe acute respiratory syndrome coronavirus 2 (SARS-CoV-2) is an enveloped, non-segmented positive-sense RNA virus belonging to the beta-coronaviridae family. This virus is known to cause severe bilateral pneumonia and acute respiratory distress syndrome (ARDS) which can lead to difficulty breathing requiring mechanical ventilation and intensive care unit management.

**Patient concerns:**

A 77-year-old female with a history of hypertension and hyperlipidemia who presented as a transfer to our hospital facility with worsening fevers, cough, and respiratory distress.

**Diagnosis:**

Chest X-rays revealed bilateral infiltrates worse at the lung bases and CT scan of the chest showed bilateral ground-glass opacities consistent with COVID-19. While our testing revealed a negative COVID-19 result at our institution, the result at a previous hospital returned a positive result.

**Interventions:**

She was being treated aggressively in the intensive care unit with high dose intravenous ascorbic acid, hydroxychloroquine, and anti-interleukin-6 monoclonal antibody. She also received a loading dose of remdesivir however was unable to complete the course due to organ failure and requirement of vasopressors for hemodynamic stability.

**Outcomes:**

She remained critically ill and was eventually placed on comfort care as per the family's wishes and passed away.

**Lessons:**

With a rapidly growing death rate and more than 200,000 confirmed cases worldwide, COVID-19 has become a global pandemic and major hit to our healthcare systems. While several companies have already begun vaccine trials and healthcare facilities have been using a wide-range of medications to treat the virus and symptoms, there is not yet an approved medication regimen for COVID-19 infections. The alarming increase in cases per day adds additional pressure to find a cure and decrease the global health burden and mortality rate.

## 1 Introduction

The novel coronavirus 2019 (COVID-19) also known as severe acute respiratory syndrome coronavirus 2 (SARS-CoV-2) is an enveloped, non-segmented positive-sense RNA virus belonging to the beta-coronaviridae family.^[1]^ COVID-19 has been found to be the cause of severe pneumonia and acute respiratory distress syndrome (ARDS) with a significantly high mortality rate.^[2]^ According to the World Health Organization, there are 207,855 confirmed cases and 8648 deaths from COVID-19 as of March 19, 2020 and rapidly increasing.^[3]^ Originating from bats like other virulent coronavirus (CoV) strains such as severe acute respiratory syndrome coronavirus (SARS-CoV) and Middle East respiratory syndrome coronavirus (MERS-CoV), COVID-19 has become the focus of the medical world and the pandemic of 2020.^[1,4]^ We present a case of elderly female presenting with fever, cough, and shortness of breath found to be positive for COVID-19 and started on high-dose IV ascorbic acid, anti-interleukin-6, hydroxychloroquine, and remdesivir requiring high ventilator settings and eventually requiring vasopressors and continuous veno-venous hemodialysis (CVVHD).

## 2 Case presentation

**Figure 1:**
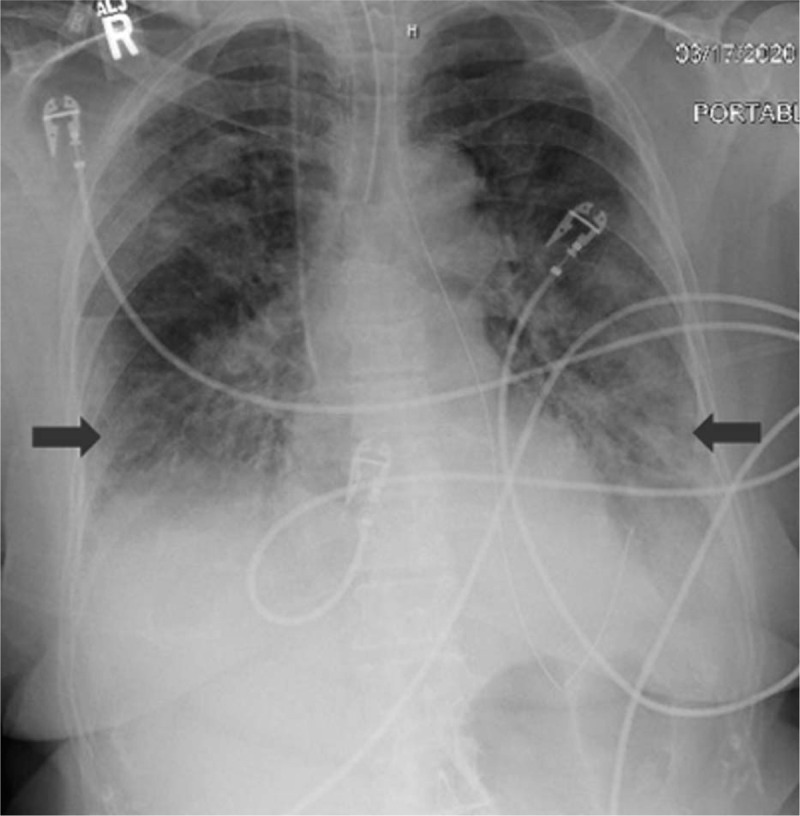
Chest X-ray showing bilateral infiltrates worsened in the lower lung fields.

**Figure 2:**
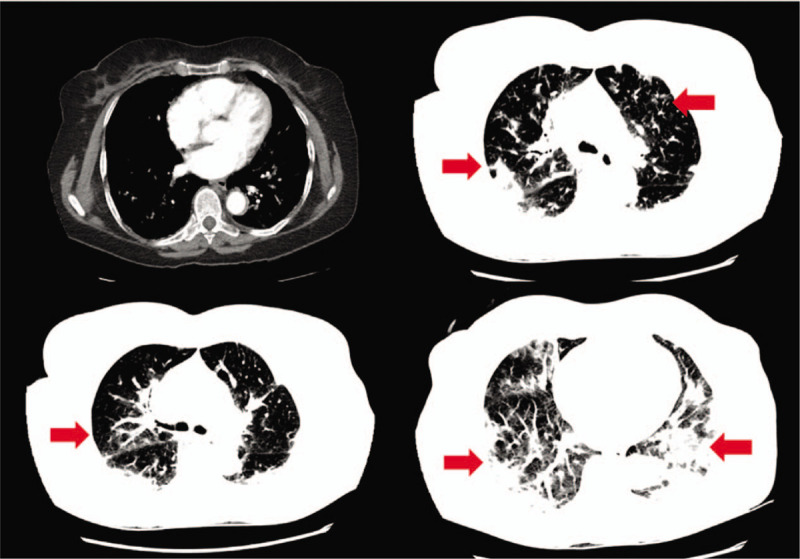
CT scan of the chest showing bilateral ground glass opacities, worsening in the lower lung fields.

A 77-year-old Middle-Eastern female with a medical history of hypertension and hyperlipidemia presented to the emergency department (ED) from a day care facility apartment where 2 people at the facility have tested positive for COVID-19 but she did not have any direct contact with these individuals. About 5 days before admission the patient developed a fever with a temperature of 102°F at home, and went to her primary medical doctor who sent her to the ED. In the ED she was found to have bilateral opacities on chest X-ray and had continued intermittent fevers with generalized weakness, cough, lethargy, and dyspnea and was sent for testing for COVID-19 then transferred to our facility for further management. In our facility, her temperature was 101.7°F, blood pressure 148/76 mm Hg, heart rate of 99 beats per minute, respiratory rate of 18 per minute, and oxygen saturation of 93% on room air. Physical exam was significant for a dry cough and bilateral rales on auscultation of the lung fields bilaterally but was unremarkable otherwise. A chest X-ray (Fig. 1) was performed showing bilateral opacities throughout the lung fields with predominance of the lower lung lobes she was admitted for possible pneumonia with isolation precautions for suspected COVID-19 and was started on oxygen via nasal cannula and on 1-gram ceftazidime intravenously every 8 hours and 500 mg azithromycin orally daily. CT scan of the chest (Fig. 2) was performed showing bilateral ground glass appearance throughout the lung with predominance in the peripheral lower lobes. Respiratory viral panel was sent including a repeat COVID-19 test (Table 1). All results came back negative however the patient's condition deteriorated 2 days after admission to our facility, and she became hypoxic to 85% oxygen saturation while on nasal cannula and remained spiking fevers up to 103.4°F. She was intubated and transferred to the intensive care unit (ICU) for further management and was switched to ceftriaxone 1 g intravenously daily and azithromycin 500 mg via orogastric tube daily and was started on hydroxychloroquine 400 mg loading dose followed by 200 mg twice daily for a 7-day course. She required 100% fraction of inspired oxygen (FiO2) and a positive end-expiratory pressure (PEEP) of 12 to maintain an oxygen saturation of >90%. 12 hours later, the COVID-19 test from the initial facility returned positive results. On day 3 of hospitalization she was started on 6 g of IV ascorbic acid twice daily and given one dose of 8 mg per kg (567 mg) of tocilizumab, an anti-interleukin-6 monoclonal antibody. Due to a shortage of vitamin C in the hospital, her dose was decreased to 1 g IV daily on the 6th day of hospitalization and she was given another dose of tocilizumab. On day 7, her PEEP increased from 12 to 16 due to worsening oxygen saturation and increased requirement despite 100% FiO2. Due to severe ARDS, the decision was made to prone the patient for 18 hours a day. She completed her course of antibiotics and hydroxychloroquine but remained on vitamin C and zinc. Approval for remdesivir was obtained from Gilead Sciences Inc and she was given a loading dose of 200 mg on day 10 and due to worsening oxygen saturation her PEEP was again increased to 18. On day 11, the patient was unable to tolerate being prone due to significant desaturation to 65% on pulse oximetry and remained supine. She eventually required levophed for maintenance of hemodynamic stability and her creatinine increased from her baseline of 0.5-0.6 since admission until day 10 to 2.65 on day 12. For this reason, remdesivir was discontinued and nephrology was consulted and recommended CVVHD on day 13. On day 14 her PEEP requirement again increased to 20 while on 100% FiO2 to maintain an oxygen saturation >90%. Her condition remained critical while being aggressively managed in the ICU and ultimately the patient's family decision was to pursue comfort measures and the patient passed away.

**Table 1:**
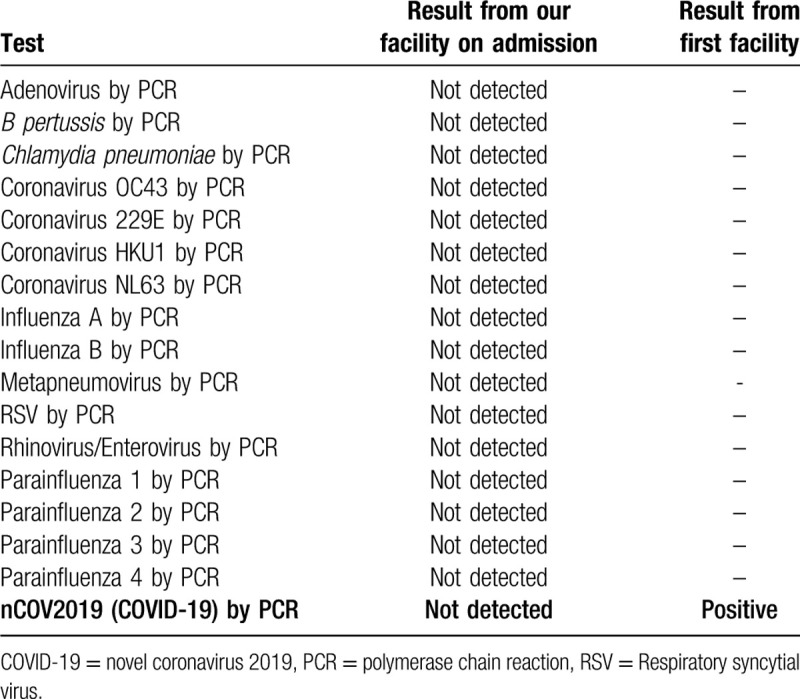
Respiratory viral panel testing by PCR.

## 3 Discussion

COVID-19 is the cause of severe viral pneumonia rapidly leading to ARDS. In a case series of 135 patients, Wan et al reported 88.9% of patients presented with a fever and 76.5% had a cough.^[5]^ Fatigue and myalgias (32.5%), headache (17.7%), and dyspnea (13.3%) were less commonly reported.^[5]^ These symptoms were also found on presentation with our patient. While the COVID-19 tests were pending, the CT scan of the chest provided valuable information as it met the trend of findings in infected patients. Wan et al obtained CT scans on all patients in their study and found bilateral involvement and multiple patchy or ground glass appearance to be the primary finding.^[5]^ Huang et al found similar findings where 98% of CT scans obtained had bilateral involvement and multilobular consolidations.^[6]^ These findings on CT scans are not unusual for a viral pneumonia. Influenza A (H1N1) was first found to cause a pandemic in 2009, a retrospective review of 92 patients by Çörtük et al found 69.6% of patients with H1N1 had bilateral patchy pneumonic infiltrates and 41.3% had bilateral ground glass opacities.^[7]^ While the lack of rapid testing for COVID-19 has caused a delay in diagnosis, perhaps the use of CT scans could provide an increased suspicion of COVID-19 infection leading to earlier treatment and management.

Our patient presented in this case received treatment with vitamin C and zinc, both of which are known to improve the human immune system and aid in shortening the duration of and improving outcomes in respiratory infections including pneumonia.^[8,9]^ In addition to vitamin and mineral supplements, hydroxychloroquine and azithromycin have obtained a large amount of attention for the treatment of COVID-19. Hydroxychloroquine, a well-known anti-malarial and auto-immune medication, is relatively inexpensive and has been extensively studied in the treatment for COVID-19. Studies have suggested hydroxychloroquine can interfere with glycosylation of the coronavirus receptors and increase endosomal pH thus inhibiting viral fusion and decreasing viral load.^[10,11]^ Gautret et al reported a synergistic effect using hydroxychloroquine and azithromycin in viral elimination and decreasing viral load.^[12]^ Despite this evidence, the use of hydroxychloroquine for viral infections has been questioned. Roques et al reported a study using chloroquine in Chikungunya virus reporting cytokines were reduced causing the adaptive immune response to be delayed, exacerbating fever, and unchanged suppression of viral load.^[13]^ While further studies are in need to provide concrete evidence on the use of hydroxychloroquine, clinical trials from China have already shown promising results for COVID-19 and several countries around the world have begun using these medications. Tocilizumab, a recombinant humanized anti-interleukin-6 receptor monoclonal antibody, has been extensively used in auto-immune conditions such as rheumatoid arthritis.^[14]^ With this monoclonal antibody, interleukin-6 function is blocked and hence the differentiation of T helper cells and B cells into immunoglobulin-secreting cells are inhibited.^[14]^ The cytokine storm observed in patients with COVID-19 has been difficult to control and manage leading to increased mortality, tocilizumab therefore helps decrease the immune response and the resulting damage caused by cytokines.^[6,15]^ While still not approved in the United States, tocilizumab has thus far shown promising results in clinical trials.^[15]^

Other treatments for COVID-19 have also emerged and have thus far shown promising results in ongoing clinical trials. Of these, remdesivir (GS-5734) and favipiravir (T-705) have become the center of attention. Remdesivir is an adenosine analog that incorporates into viral RNA causing premature termination.^[10,14]^ It has been found effective at inhibiting viral replication in Ebola, SARS-CoV, and MERS-CoV infections.^[10,16,17]^ Favipiravir, an RNA-dependent RNA polymerase inhibitor, has already obtained approval for the treatment of COVID-19 in China on February 15th, 2020.^[18]^ Studies have shown favipiravir inhibited RNA polymerase activity and thus prevented replication of RNA viruses like COVID-19 with minimal side effects.^[18]^ Remdesivir (GS-5734, Gilead Sciences Inc.) is currently under several clinical trials and all of its side effects have not yet been defined. In our patient, within 2 days of starting remdesivir our patient had worsening renal function eventually requiring CVVHD and vasopressors thus preventing further treatment with the medication. While our patient was critically ill in the ICU, it is not known if this medication was the cause for further decompensation due to kidney injury. Further studies and clinical trials are required to fully understand the role of remdesivir and other medications in COVID-19 infected patients.

## 4 Conclusion

COVID-19 is a serious infection that has led to thousands of cases of severe pneumonia, ARDS, and even deaths across the globe. As of now there are no approved treatments for this viral pandemic. While several medications have shown to be effective in clinical trials, further studies are needed to establish dosing, treatment course, and side effects of these medications. As the number of cases and deaths continue to increase in the world, the race to develop faster testing modalities to rapidly diagnose and manage these patients earlier continues to be the focus of the global healthcare system.

## Author contributions

**Conceptualization:** Steven Douedi, Jeffery Miskoff.

**Writing – original draft:** Steven Douedi.

**Writing – review & editing:** Steven Douedi, Jeffery Miskoff.
